# The standard operating procedure of the DOE-JGI Microbial Genome Annotation Pipeline (MGAP v.4)

**DOI:** 10.1186/s40793-015-0077-y

**Published:** 2015-10-26

**Authors:** Marcel Huntemann, Natalia N. Ivanova, Konstantinos Mavromatis, H. James Tripp, David Paez-Espino, Krishnaveni Palaniappan, Ernest Szeto, Manoj Pillay, I-Min A. Chen, Amrita Pati, Torben Nielsen, Victor M. Markowitz, Nikos C. Kyrpides

**Affiliations:** Genome Biology Program, Department of Energy Joint Genome Institute, 2800 Mitchell Drive, Walnut Creek, USA; Biosciences Computing, Computational Research Division, Lawrence Berkeley National Laboratory, 1 Cyclotron Road, Berkeley, USA; Present Address: Computational Biology Group, Celgene Corporation, Summit, USA

**Keywords:** Microbial Genome Annotation, SOP, IMG, JGI

## Abstract

The DOE-JGI Microbial Genome Annotation Pipeline performs structural and functional annotation of microbial genomes that are further included into the Integrated Microbial Genome comparative analysis system. MGAP is applied to assembled nucleotide sequence datasets that are provided via the IMG submission site. Dataset submission for annotation first requires project and associated metadata description in GOLD. The MGAP sequence data processing consists of feature prediction including identification of protein-coding genes, non-coding RNAs and regulatory RNA features, as well as CRISPR elements. Structural annotation is followed by assignment of protein product names and functions.

## Introduction and Requirements

The DOE-JGI Microbial Genome Annotation Pipeline performs structural and functional annotation of bacterial and archaeal genomes included into the Integrated Microbial Genome (IMG) system [1]. Annotation consists of the identification of RNA and protein-coding genes and repeats, as well as the prediction of functions for each gene (product name assignment). The annotated microbial genomes datasets produced by the MGAP are integrated into IMG, where they can be analyzed or manually edited in the context of a comprehensive set of publicly available genomes.

Compared to the previous version, the pipeline contains several new steps (e.g. the addition of several modules that form a data preprocessing track), some tool replacements (e.g. Prodigal is now used for *ab intio* gene prediction instead of GeneMark and Metagene Annotator), as well as updated software and models (e.g. the rRNA Hidden Markov Models). So far this version of the pipeline has processed more than 800 microbial genomes with at least 10 new ones being processed on a daily basis. While no comprehensive reannotation of all genomes processed by the older version of the pipeline has been planned, point updates of the features affected by the pipeline changes are under way. Some of them, including CRISPR annotations and Pfam and TIGRfam assignments, have been finalized. The MGAP requires a multi-FASTA file of assembled nucleotide sequences as an input for gene calling. In addition, each sequence dataset submitted for annotation needs to be associated with an analysis project that has already been specified in the Genomes OnLine Database [2]. Microbial genome annotation consists of three stages: sequence data preprocessing, feature prediction, and functional annotation. Feature prediction (which includes gene prediction and repeat identification) produces a GenBank file that does not have any functional information for the predicted genes. Subsequently, these genes are assigned functions and are integrated into IMG.

## Procedure and Implementation

The MGAP stages and individual steps are further described below.

### Sequence data preprocessing

**Fig. 1:**
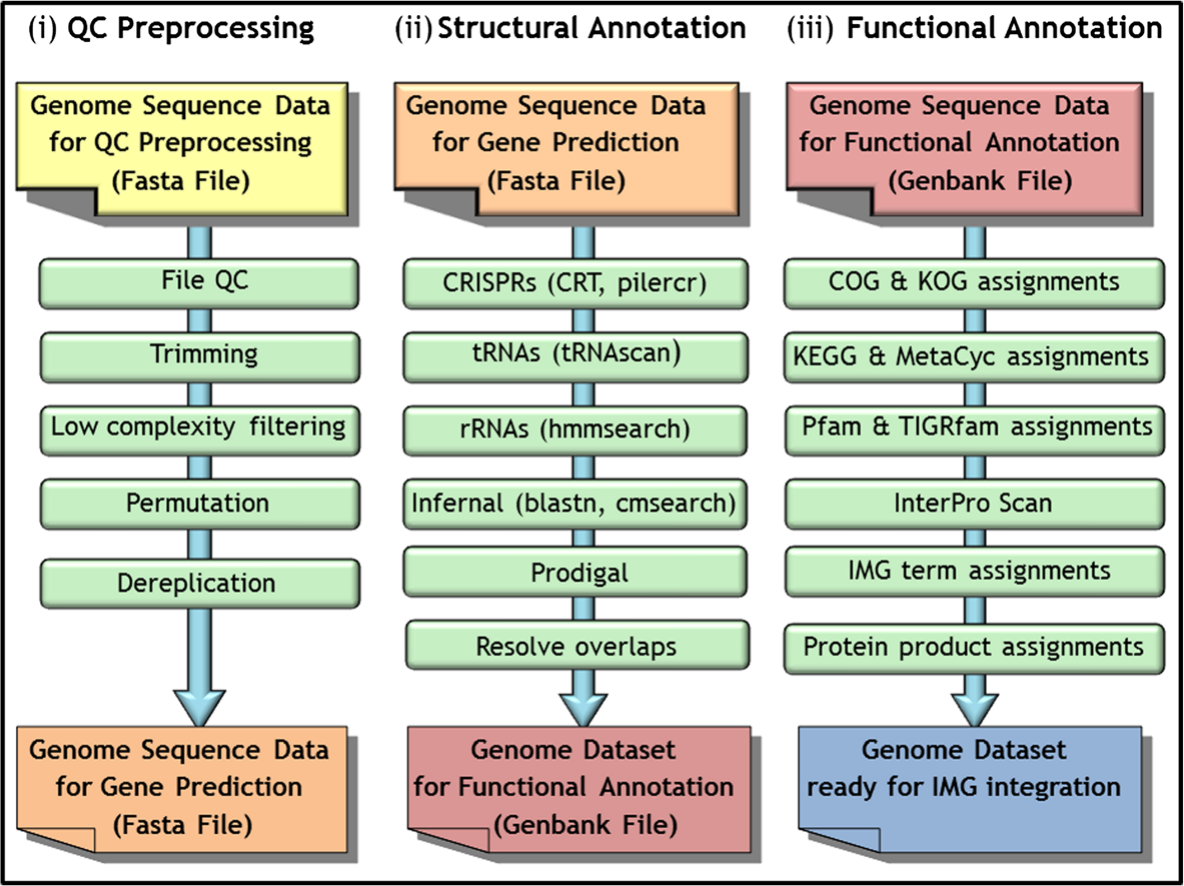
Genome sequence data preprocessing (**i**), structural (**ii**) and functional annotation (**iii**) steps of the MGAP v.4

All genome datasets undergo preprocessing in order to ensure that only good quality sequences are processed in the gene prediction stage, as illustrated in Fig. 1(i). First, all ambiguous nucleotides in the sequence datasets are replaced by N’s and sequences with characters that do not belong to the {A,C,G,T,N} set are not considered further. Additionally, the headers in multi-FASTA files are changed to ensure that all contig and scaffold names are unique and compatible with the tools employed in subsequent stages. The pipeline creates a mapping file, which records the correspondence between old and new sequence headers. Furthermore, sequences shorter than 150 nt are removed. Second, the sequences are trimmed in order to remove trailing ‘N’s. The trimmed sequences then have to pass a low complexity filtering where low complexity noisy sequences are identified using the DUST [3] application and eliminated. Finally, for finished circular genomes the pipeline attempts to detect the origin of replication by running BLASTx against an in-house curated database of genes located near the origin of replication. If an origin of replication is successfully detected, the sequence is permuted so that it starts at that position.

### Feature prediction

As illustrated in Fig. 1(ii), feature prediction starts with the detection of CRISPR arrays, followed by non-coding RNA genes (tRNA, rRNA and other RNA genes), and finally the prediction of protein coding genes.

CRISPR elements are identified using the programs CRT [4] and PILER-CR v1.06 [5]. For PILER-CR the maximum spacer length is set to 100 and the CRISPR element needs to have at least 5 repeats, which have at least 90 % identity to each other and at least 75 % identity to the consensus sequence of the repeat. For CRT the pipeline runs a modified version of the latest official CRT-CLI 1.2 version. Specifically, the modified CRT has the capability to read multi-FASTA files, detect truncated repeats at the ends of the contigs/scaffolds and deal with spacer artifacts and repeats that contain Ns. This version also executes checks for repeat and spacer length ratios, while the length and similarity checks are performed as part of “all vs. all” spacer and repeat comparisons. Additionally, the progression step of the sliding search window was reduced to 1, while threshold values and search ranges, which were strictly defined in the original software, can be changed from default values on the command line together with the new options and arguments.

In our modified CRT the default values for the minimum and maximum repeat lengths are set to 20 and 50 bp, respectively, while the minimum and maximum spacer lengths are set to 20 and 60 bp, respectively. The ratio of the spacer lengths to the repeat lengths have to be between 0.6 and 2.5. The search window is 7 bp long and an element needs to have at least 3 repeats that have a minimum of 70 % identity. The predictions from PILER-CR and CRT are concatenated and, in case of overlapping predictions, the CRT prediction is retained.

Protein-coding genes and non-coding RNA genes are identified using a combination of Hidden Markov Models and sequence similarity-based approaches. The first category of non-coding RNAs, tRNAs, are predicted using tRNAscan-SE 1.3.1 [6], and the best scoring predictions are selected. Ribosomal RNA genes (5S, 16S, 23S) are predicted using hmmsearch tool from the package HMMER 3.1b2 [7] and a set of in-house curated HMMs derived from an alignment of full-length rRNA genes selected from IMG isolate genomes. Both tRNAscan-SE and hmmsearch use a domain-specific set of models for Bacteria and Archaea, which is selected based on the taxonomic information provided in the corresponding GOLD Analysis Project.

MGAP also predicts other non-coding RNAs and regulatory RNA features, such as riboswitches. With the exception of tRNAs and rRNAs, all models from Rfam 10.1 [8] are used to search the genome sequences. For faster detection, sequences are first compared to a database containing all the ncRNA genes and other RNA features in the Rfam database using BLASTn, with a very loose e-value cutoff (1.0e −10). Subsequently, contigs/scaffolds with hits to this database are searched against Rfam covariance models using the program cmsearch from the INFERNAL 1.0.2 package [9].

The identification of protein-coding genes is performed using the Prodigal v2.6.2 *ab initio* gene prediction program [10]. Overlaps between predicted features of different type (e. g. ncRNAs and protein-coding genes) get resolved based on an in-house curated set of rules. Every annotated gene is assigned a locus tag of the form PREFIX_SUFFIX where the prefix is the identifier of the GOLD Analysis Project associated with the genome dataset and the suffix is a number that identifies a certain gene on a particular sequence. This assignment scheme guarantees that each gene within a sequencing project gets a unique locus tag. The output of this stage is a GenBank format genome data file.

Every GenBank file by the pipeline or submitted by a user, must pass an additional validation step before it is forwarded to the next stage for functional annotation. The validation involves checking the file format, matching the gene coordinates to their translations and sequence lengths. The validation also removes phage PhiX sequences, which can be found as a common contaminant in isolate genomes sequenced with Illumina technology [11]. These are identified by running BLASTn against the PhiX genome sequence with an e-value of 0.01 and 90 % identity. If a hit covers 80 % or more of a query contig/scaffold, the latter gets removed. As a final step the validation script also assesses the quality of a genome, which determines whether it will be included as a reference genome for taxonomic comparisons with other genomes and metagenomes. A genome could get marked as “low quality” and excluded from taxonomic reference database if (a) it lacks phylum-level taxonomic assignment or (b) its coding density (defined as total length of nucleotide sequence of predicted genes divided by the total length of nucleotide sequence) is less than 70 % or greater than 100 % or (c) there are more than 300 sequences per million base pair or (d) the number of genes per million base pair is less than 300 or greater than 1200. These values were set after manual analysis and benchmarking and are intended to prevent highly fragmented genomes and/or genomes with high rate of sequencing artifacts from being included in a taxonomic reference database.

### Functional annotation

After a genome dataset undergoes structural annotation, the resulting protein-coding genes are compared to protein families and the proteome of selected “core” genomes which are publicly available, and a protein product name is assigned to each gene as discussed below and illustrated in Fig. 1(iii).

#### Protein families

1. COG & KOG assignment: protein sequences are compared to COG PSSMs obtained from the CDD database [12] using the program RPS-BLAST at an e-value cutoff of 1e–2, with the top hit retained. The alignment length needs to be at least 70 % of the consensus sequence length.
2. KEGG Orthology term assignment: Genes are associated with KO terms [13] as follows. First, the genes that can be unambiguously mapped to the entries in KEGG Genes database are assigned the KO terms associated with the corresponding KEGG gene. The gene to KEGG gene mapping is based on NCBI’s GI numbers and GeneIDs. For genes that are not mapped to KEGG genes, USEARCH is run against the database of KEGG genes by applying UBLAST [14]. The results of this search are organized in a list of candidate KO assignments. KO terms are assigned to genes using a subset of this list, whereby the threshold is defined by an E-value cutoff of 1e–5, KO assignments are selected from the top 5 hits, with 30 % or better alignment sequence identity, and alignment percentage of at least 70 % over the length of the query gene and KEGG subject gene.
3. MetaCyc assignment: genes are associated with MetaCyc [15] reactions as follows. First, genes are mapped to KO terms as described above, whereby KO terms are associated with Enzyme Commission numbers using the KEGG KO term to Enzyme relationship provided by KEGG. Next, genes are associated with MetaCyc reactions via EC numbers.
4. Pfam & TIGRfam assignments: protein sequences are searched against Pfam [16] and TIGRfam [17] databases using HMMER 3.0. For TIGRfam, the noise cutoff (−−cug_nc) is used, with hits below the trusted cutoff and at/above the noise cutoff flagged as “marginal”. For Pfam, the gathering threshold (−−cut_ga) is used inside the pfam_scan.pl script. The script also helps resolving overlaps between hits to Pfam models from the same clan in order to generate final Pfam assignments.
5. InterPro Scan: Additional protein family annotations for SMART, PrositeProfiles, PrositePatterns, and SuperFamily are provided by InterPro Scan (run with default parameters) [18].

#### IMG terms

IMG terms [19] were introduced with the goal of addressing problems related to the consistency of functional annotations in IMG. IMG terms model the relationship between the protein product of a gene and its mature, fully functional form. Accordingly, three types of IMG terms have been introduced: *protein product*, *modified protein* and *protein complex*. Initial assignment of genes to IMG terms is done manually, whereby each IMG term is functionally coherent and is not required to contain sequences that share similarity. These IMG terms are recorded in a BLAST-able database that is used for propagating the terms to other genes following three complementary methods applied in succession:

1. Method 1 is based on the BLAST-able database of gene sequences that have been associated manually with IMG terms. Gene sequences of new genomes are compared against this database: a gene *g* with hit to a sequence associated with IMG term *t* satisfying the following criteria will be assigned this IMG term: top hit, e-value < = 1e-5, > = 90 % identity, alignment > = 80 % on both query and subject sequences, smallest to largest sequence length ratio of query and subject sequence > = 70 %.
2. Method 2 is applied to genes that are not assigned an IMG terms using Method 1 and is based on a set of rules devised by domain experts at JGI for mapping functional annotations (COG, Pfam, TIGRfam) to IMG terms. An example of such a rule is: “assign IMG Term 6 (*replicative DNA helicase loader DnaB*) to a gene if the gene is annotated with COG3611 (*replication initiation/membrane attachment protein*)”.
3. Method 3 is applied to genes that are not assigned an IMG term using Methods 1 and 2, and is based on gene bi-directional best hits (BBHs) across all genomes available in IMG. For a gene *g*:
  - Get *g*’s top 5 BBH genes satisfying the following conditions: sequence alignment length > = 70 %, percent identity > = 25 %. No IMG terms can be assigned to gene *g* unless there are at least 5 BBH genes satisfying these conditions.
  - Let *Set T* be the set of all manually assigned IMG terms (i.e., not automatically populated terms) of any of the 5 BBH genes above. Check each term T1 in *Set T*: (i) if the 5 BBH genes have conflicting term assignments (e.g., some were assigned term T1, while others were assigned term T2), then no terms in *Set T* can be assigned to gene *g*; (ii) if there are no conflicting IMG term assignments and at least 2 of the 5 BBH genes are associated with term T1, then assign T1 to gene *g*; (iii) if there are no conflicting IMG term assignments but fewer than 2 of the 5 BBH genes are associated with the same IMG term, then repeat this step with top 10 BBH genes.

Bi-directional best hits are computed between protein sequences of pair of genomes using USEARCH by applying UBLAST with a nominal e-value cutoff of 1e–2. An effective database size (−ka_dbsize 700,000,000) is used in order to make the e-values comparable across pairs of genome computations.

#### Protein product names

There are two gene product names associated with every gene in IMG: (i) original gene product name, which is included from the original genome datasets archived at GenBank or submitted by users for inclusion into IMG without the IMG product name assignment; (ii) IMG product name, generated by the Inative product name assignment procedure of the IMG described below. Every gene in IMG with IMG product name is associated with an “IMG Product Source” attribute, which specifies the source of the IMG assigned product name. The value of this field can be:

- ITERM:xxxxx: The source of the gene product name is its associated IMG term xxxxx;
- TIGRxxxxx: The source of the gene product name is its associated TIGRfam xxxxx;
- COGxxxx: The source of the gene product name is its associated COGxxxx;
- PFAMxxxx: The source of the gene product name is the associated PFAMxxxx.

IMG Protein product names are assigned to genes in two stages: (i) product names are assigned based on IMG terms whenever they are available, (ii) if IMG terms are not available then protein family associations for genes in individual genome datasets are employed for assigning product names. Protein product names assignments based on IMG terms rely on protein sequence similarities between the genes of the new dataset and genes of all other genomes in the IMG data warehouse.

The IMG protein product of a gene *g* is assigned as follows:

1. If gene *g* is associated with one or more IMG terms, then the IMG term(s) becomes the new IMG product name(s) of *g*.
2. For genes that are not associated with IMG terms based on Methods 1–3 described above, and therefore do not have a product name based on IMG terms, assignment of TIGRfam names as product names is attempted: the gene without a product name is assigned a name of a TIGRfam if it has a TIGRfam hit. If a gene has a hit to only one TIGRfam, the name of this TIGRfam is assigned; if more than 1 TIGRfam is assigned, the name of a TIGRfam of the type “equivalog” is assigned.
3. For genes that were not associated with a product name using IMG terms or TIGRfam names, product names are assigned based on the name of their COG hit. If the COG name is “uncharacterized conserved protein” or contains “predicted”, the name has the format “COG.cog_name, COG.cog_id”.
4. For genes that were not associated with a product name using IMG terms, TIGRfam or COG names, product names are assigned based on the name of their Pfam hit, where the product name is a concatenation of Pfam family description (attribute “description” in pfam_family) with “protein”. If a protein has hits to multiple Pfams, their descriptions are concatenated with “/” as a separator and a word “protein” added in the end.

A translation table for protein product names based on IMG terms, TIGRfam, COG and Pfam descriptions is employed in order to ensure that the product names are compatible with GenBank requirements when the datasets are submitted to GenBank.

#### Functional annotation sources

- COG 2014 Version (November 2014)
- KEGG Release 71.0, July 2014
- MetaCyc Release 18.1, June 2014
- PFAM 28.0, May 2015
- TIGRfam Release 14.0, January, 2014
- InterPro Data Release 48

#### Prediction of protein sequence features

Signal peptide feature prediction employs SignalP 4.1 [20]. The model used is determined by the gram stain annotation field for the genome (gram+, gram-, Euk). If the gram stain field is not specified in the IMG data, assume it’s gram- (gram negative).

Transmembrane helices are predicted using TMHMM2.0c [21].

Both tools get executed with the default parameters.

## Discussion

The DOE-JGI MGAP performs annotation for bacterial and archaeal genomes. The pipeline consists of custom scripts and publicly available tools. Consistency and reproducibility of the results produced by the MGAP depend on the tools and annotation resources used in the pipeline. Thus, updated versions of resources such as Rfam, Pfam, and KEGG may improve the breadth and depth of functional annotations.

In order to apply the DOE-JGI MGAP on their datasets, users need to first specify their analysis projects in GOLD and then provide their genome datasets via IMG’s data submission site.

We will continue to extend the MGAP with the goal of improving the identification and characterization of genes in the genome datasets it processes.
