# Erratum to: The standard operating procedure of the DOE-JGI Microbial Genome Annotation Pipeline (MGAP v.4)

**DOI:** 10.1186/s40793-016-0148-8

**Published:** 2016-03-21

**Authors:** Marcel Huntemann, Natalia N. Ivanova, Konstantinos Mavromatis, H. James Tripp, David Paez-Espino, Krishnaveni Palaniappan, Ernest Szeto, Manoj Pillay, I-Min A. Chen, Amrita Pati, Torben Nielsen, Victor M. Markowitz, Nikos C. Kyrpides

**Affiliations:** Department of Energy Joint Genome Institute, Genome Biology Program, 2800 Mitchell Drive, Walnut Creek, USA; Biosciences Computing, Computational Research Division, Lawrence Berkeley National Laboratory, 1 Cyclotron Road, Berkeley, USA; Present Address: Computational Biology Group, Celgene Corporation, Summit, USA

After publication of this study [1], we noticed that the Strain ID summary was not removed from the PDF due to a copyediting error. The original version of this article was corrected. The publisher apologizes for any inconvenience caused.
